# Automated Classification and Cluster Visualization of Genotypes Derived from High Resolution Melt Curves

**DOI:** 10.1371/journal.pone.0143295

**Published:** 2015-11-25

**Authors:** Sami Kanderian, Lingxia Jiang, Ivor Knight

**Affiliations:** Canon U.S. Life Sciences, Rockville, MD, United States of America; University of New England, AUSTRALIA

## Abstract

**Introduction:**

High Resolution Melting (HRM) following PCR has been used to identify DNA genotypes. Fluorescent dyes bounded to double strand DNA lose their fluorescence with increasing temperature, yielding different signatures for different genotypes. Recent software tools have been made available to aid in the distinction of different genotypes, but they are not fully automated, used only for research purposes, or require some level of interaction or confirmation from an analyst.

**Materials and Methods:**

We describe a fully automated machine learning software algorithm that classifies unknown genotypes. Dynamic melt curves are transformed to multidimensional clusters of points whereby a training set is used to establish the distribution of genotype clusters. Subsequently, probabilistic and statistical methods were used to classify the genotypes of unknown DNA samples on 4 different assays (40 *VKORC1*, CYP2C9*2, CYP2C9*3 samples in triplicate, and 49 *MTHFR c*.*665C>T* samples in triplicate) run on the Roche LC480. Melt curves of each of the triplicates were genotyped separately.

**Results:**

Automated genotyping called 100% of *VKORC1*, CYP2C9*3 and MTHFR c.665C>T samples correctly. 97.5% of CYP2C9*2 melt curves were genotyped correctly with the remaining 2.5% given a no call due to the inability to decipher 3 melt curves in close proximity as either homozygous mutant or wild-type with greater than 99.5% posterior probability.

**Conclusions:**

We demonstrate the ability to fully automate DNA genotyping from HRM curves systematically and accurately without requiring any user interpretation or interaction with the data. Visualization of genotype clusters and quantification of the expected misclassification rate is also available to provide feedback to assay scientists and engineers as changes are made to the assay or instrument.

## Introduction

High Resolution Melting (HRM) is used in combination with PCR as a method for differentiating genotypes based on the disassociation of DNA binding dyes [1]. Without additional intervention or modification of the chemistry, after PCR, the temperature is raised slowly while fluorescence measurements are made. During this process, double strand DNA separates and the dye is released, decreasing fluorescence. A plot of relative fluorescence versus temperature constitutes a melt curve [2–4]. This phenomenon is used to identify of Single Nucleotide Polymorphisms (SNPs) whereby melt curves can be distinguished from each other when using instrumentation capable of high resolution temperature and fluorescence measurement. Melt curves of different genotypes can be distinguished based on differences in their shapes and/or relative melting temperature shift due to different bond strengths between different base pairs between complementary strands and stacking between adjacent bases that are dependent on salt concentrations [5]. However, melt curves of the same genotype do not overlay perfectly due to temperature variability from run to run or across a well plate [6–8], slight differences in instrumentation or chemistry components. As long as the melt curve variability between genotypes is greater than the variability within genotypes, the classification of different genotypes is possible. In order to enhance the separation of different melt curves, curves are typically either subtracted from a positive control to obtain difference plot or various transformations of the data, such as the negative derivative of fluorescence versus temperature, are computed [3].

Currently, software exists that performs genotyping and clustering, but not without user interaction [9–11]. This interaction includes instances whereby a user selects different temperature windows before and after the rapid rate of change in fluorescence in order to determine and remove background fluorescence. Determination of the background signal can vary depending on the window locations selected by the operator, which may consequently alter the genotype classification. Systematic and fully automated genotyping analysis software would be advantageous; particularly under high throughput conditions where analytical results must be consistent no matter which operator uses the software.

Here we describe an automated method whereby the probability that a DNA sample belongs to each possible known genotype is computed. Furthermore, if so desired, users can visualize genotype clusters in two dimensions whereby each melt curve is plotted as a point and the positioning of each point or sample has a physical meaning. Although a method for determining the likelihood of each possible genotype derived from cluster plots has been reported [12], the methods and assumptions made in implementing its approach are different from ours and the location of each plotted point lacks physical meaning with respect to a fixed coordinate system.

## Materials and Methods

### Clinical samples and data sets

HRM curves from 4 different assays were analyzed. 3 assays were small amplicon Warfarin sensitivity assays:

*VKORC1 c*.*1639G>A*, CYP2C9*2 c.*430C>T*, CYP2C9*3 c.*1075A>C*, and one assay was a snapback primer coagulation factor assay: *MTHFR c*.*665C>T*, associated with thrombophilia. The Primer sequences, amplicon size, and SNP are listed in Table 1.

**Table 1:** Assay information including Target, amplicon size, and primer sequences.

Target	Amplicon Size (bp)	Forward Primer Sequence (5' to 3')	Reverse Primer Sequence (5' to 3')
*VKORC1 c*.*1639G>A*	63	CAAGAGAGAGCCTGAAAAACAACCATTG	TGCTAGGATTATAGGCGTGAGCC
CYP2C9*2 c.*430C>T*	123	GAATTTTGGGATGGGGAAGAG	TCCAGTAAGGTCAGTGATATGG
CYP2C9*3 c.*1075A>C*	44	TGGTGCACGAGGTCCAGA	GCTGGTGGGGAGAAGGTCA
*MTHFR c*.*665C>T*	103	GAGGCTGACCTGAAGCACTTG	CAGGGAGCCGATTTCACCTTCACAAAGCGGAAGAATGTGTC

All clinical samples were provided by Associated Regional and University Pathologists (ARUP) Laboratories and protocols were approved by the University of Utah Institutional Review Board, IRB #00007740 entitled, “Blood, Urine, Stool, or Saliva Samples for Validation of Assay Methods Used in Clinical Testing”. The purpose of this IRB is to allow validation of assays using ARUP samples that have been anonymized and de-identified. ARUP is wholly owned by the University of Utah. A medical director of ARUP and professor at the University of Utah arranged for the samples under a grant from Canon to the University of Utah. The IRB waived the consent requirement because DNA samples were anonymized and de-identified at ARUP before receipt as a requirement of the IRB. These samples were likely whole genome amplified at the University of Utah before they were sent to Canon.

Samples were selected to cover all possible genotypes and were not representative of the allele frequency in the general population. 50 ng of DNA was used for testing which yielded an enriched concentration of 10ng/μL determined by UV spectrometry (Nanodrop1000). All PCR and melting assays were conducted using a Roche LightCycler480 in a 96 well plate format.

### PCR Protocols

Warfarin assays were amplified in 20 μL reaction volume in Canon buffer (50 mM Tris pH 8.0, 50 mM KCl, 0.01 mM EDTA, 1 M Betaine, 0.04% Tween 20, and 2% DMSO), 2 mM MgCl_2_, 1U Takara Ex TaqTM DNA Polymerase, Hot-Start Version (TaKaRa Bio USA); 1x LCGreen Plus (BioFire Diagnostics), 0.5 μM forward and reverse primers, 0.2 mM dNTPs, and 2.5 ng/μL human genomic DNA The amplification protocol included an initial denaturation at 95°C for 1 min, followed by 40 cycles at 95, 62, and 72°C for 5 s at each step; cooled to 37°C for 10 s, heated to 95°C for 10 s, prior to running a melt from 55–95°C with 20 data samples acquired per degree at a ramp rate of 0.03°C/s.

Snapback PCR products were generated for MTHFR c.665C>T. Asymmetric PCR was performed in triplicate with 0.1 μM of the forward primer, 0.5 μM of the Snapback reverse primer, 0.2 mM each dNTP, 1x LC Green Plus, 0.1 U/μL of Takara Ex Taq DNA Polymerase, Hot-Start Version (TaKaRa Bio USA), and 3 mM MgCl2 in Canon buffer described above in a 10 μL reaction volume. PCR cycling consisted of an initial denaturation at 95°C for 2 min, followed by 60 cycles of 95°C for 10 s, 58°C for 10 s, and 76°C for 15 s. To facilitate the snapback molecule formation, PCR products were subsequently denatured by heating to 95°C for 10 s and cooled to 45°C for 1 s prior to running a melt from 45°C–95°C with 10 data samples acquired per degree at a ramp rate of 0.06°C/s.

For each assay, a training set of known genotypes was obtained for supervised machine learning. The training set for each assay consisted of one plate of 32 DNA samples run in triplicate for a total of 96 samples. Then genotype classification of DNA samples from a subsequent blinded data set (cross-validation) was performed on 40 DNA samples for each of the Warfarin assays and 49 DNA samples for the MTHFR c.665C>T locus run in triplicate for a total of 120 melt curves and 147 melt curves respectively. The true genotype of each sample was determined by Sanger sequencing. The accuracy of the cross-validation determines the true unbiased performance of the software in conjunction with the system as a whole.

### Automated genotyping procedure

**Fig 1:**
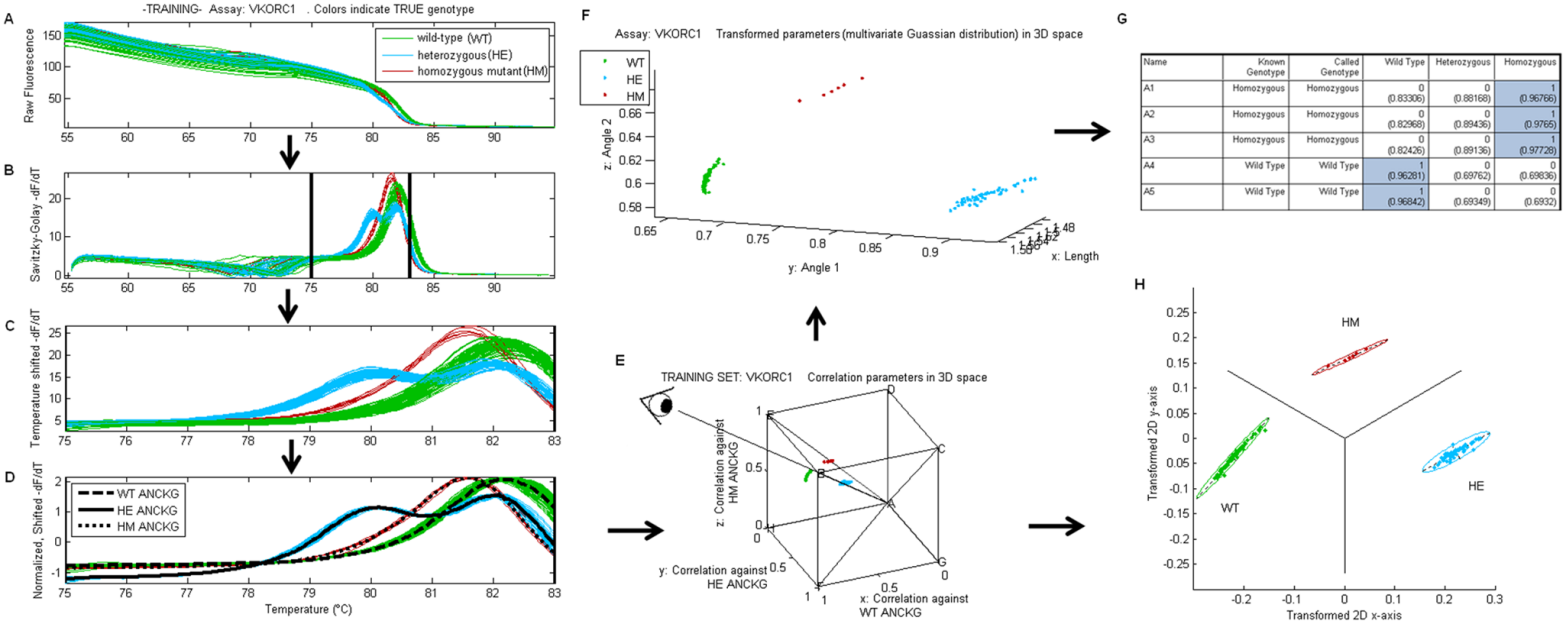
Automated genotyping procedure. A. Fluorescence (*F*) versus temperature (*T*). B.–*dF/dT* versus *T*. C. Temperature shifted–*dF/dT*. D. Normalized–*dF/dT* curves with training set genotype averages (black lines). E. A 3D point represents each curve correlated against each average curve. F. Points transformed to spherical coordinates. G. Genotype likelihood table H. 2D projection of correlation parameters for visualization.

The automated genotyping procedure follows a series of steps as shown in Fig 1. The training procedure using melt curves of known genotypes is shown in the flowchart of S1 Fig. The genotyping procedure of melt curves of unknown genotypes is shown in the flowchart of S2 Fig. Software was developed in MATLAB 6.1 (The MathWorks Inc., Natick, MA). Once finalized; it was re-written and compiled in C/C# as a standalone application.

### Computation of the derivative of fluorescence

First, raw fluorescence data (Fig 1A) were resampled by interpolation to have fluorescence readings at equally spaced temperature intervals. A Savitsky-Golay FIR filter [13,14] with fixed coefficients was subsequently convoluted with the florescence values to obtain the negated derivative of fluorescence with respect to temperature (Fig 1B). A 1°C window size and second polynomial order used to generate filter coefficients. Only data within a certain temperature range is used in subsequent analysis. The range is automatically determined by the software in order to maximize the separation of different genotype clusters, more specifically to minimize the misclassification rate, and is described in the S1 Text. The temperature range of the VKORC1 example shown in Fig 1 is from 75 to 83°C.

### Temperature shifting and data normalization

**Fig 2:**
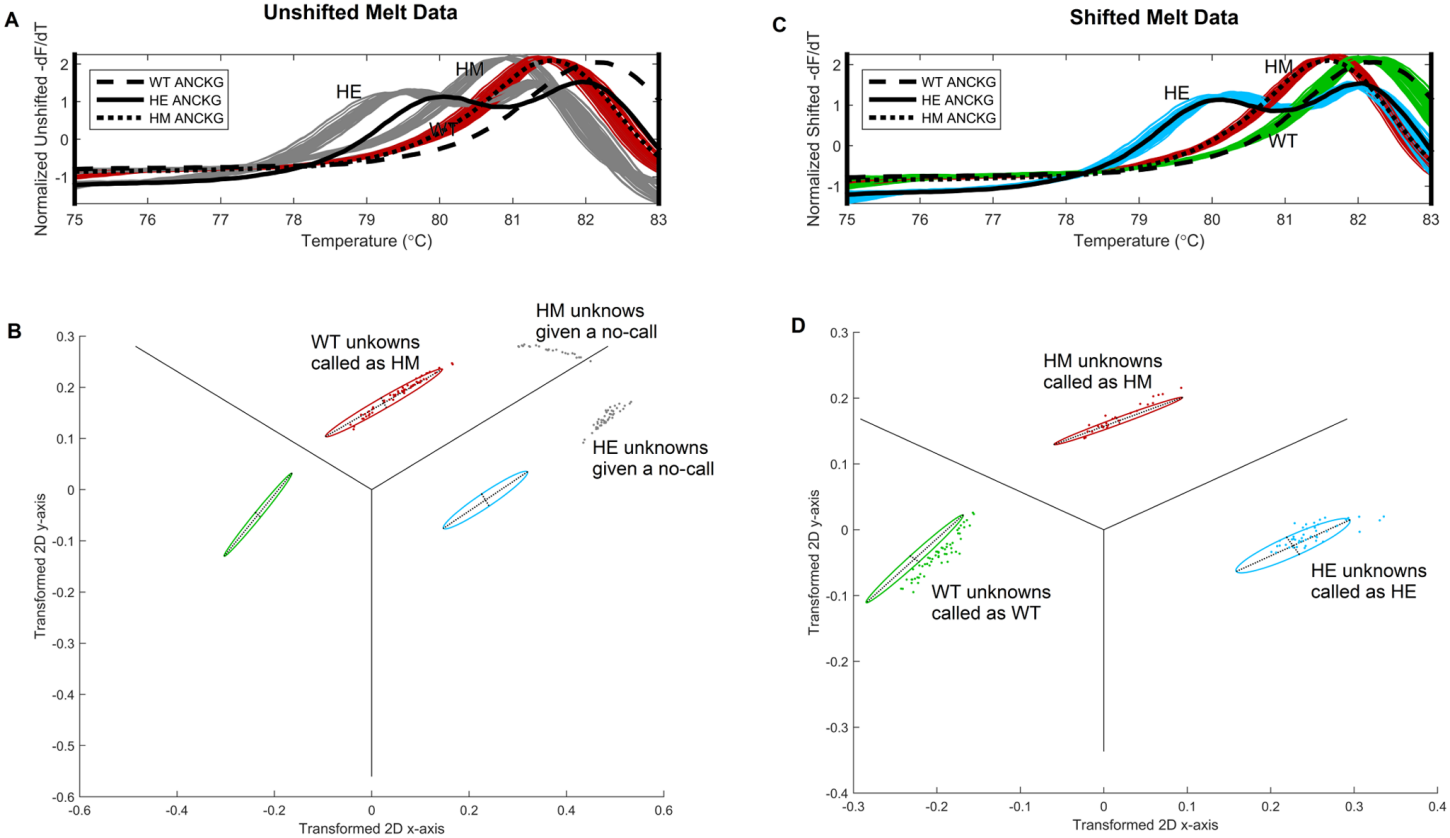
A. Cross validation normalized curves and training set genotype averages (black lines). Without temperature shifting, wild-types are called as homozygous mutants (red), homozygous mutants and heterozygotes are given “no calls” (gray). B. Corresponding 2D cluster visualization. C. With temperature shifting, all calls are correct. D. Corresponding cluster plot showing overlap between validation set (points) and training set (ellipses).

Data was collected from multiple well plates, where each well plate had two or three positive controls. Positive controls from each well plate were averaged and the average positive control from the first training set plate was used as reference. Subsequent plates contained the same positive controls usually in the same wells. The derivative curve of the positive control was then shifted by various degrees until it correlated maximally with the positive control of the reference well plate as shown in Fig 2. This was to control for shifts in measured temperature that may occur between well plate runs. In these experiments, wild-type DNA was used as positive controls. Once the optimal shift was determined, all melt curves from the same well plate were shifted by this amount. Afterwards, each melt curve derivative is normalized to have zero mean and unit standard deviation as shown in Fig 1D. The importance of the use of positive controls for temperature shifting is shown in Fig 2.

### Obtaining Averaged Normalized Curve for each Known Genotype (Training set only)

A DNA training set was required for each assay where all curves of the same genotype were averaged to get an Averaged Normalized Curve of Known Genotype (ANCKG) as shown by the black lines in Fig 1D.

### Computation of correlation parameters and spherical coordinates

Each individual melt curve derivative was correlated against each ANCKG. Thus when there are three possible genotypes, each melt curve is represented by a point in 3D space as shown in Fig 1E:

$$\text{Correlation vector}:\;r=\left[\begin{array}{c} r_{1}\\ r_{2}\\ \vdots \\ r_{Nc} \end{array}\right]$$ (1)

If there are *N*_*c*_ number of known classes or genotypes, the dimensions of the vector ***r*** are *N*_*c*_x1. For all assays tested here, *N*_*c*_ = 3. *N*_*c*_ can be greater than 3 in multiplex cases where searching for SNPs in more than one base pair.

For a given genotype, the distribution of correlation coefficients of a set of dynamic curves with their averaged normalized curve is not normally distributed (see S3 Fig). Probability calculations of parameters that are normally distributed are easily calculated, as equations that describe their distributions are known. For this reason the correlation vector of parameters for each dynamic curve was transformed into spherical coordinates consisting of a length parameter, *l* and (*N*_*c*_-1) angle parameters as follows:

$$\text{length: }l=\sqrt{\sum_{i=1}^{Nc}{\left(r_{i}\right)}^{2}}$$ (2)

$$k^{th}\text{ angle: }a_{k}={\text{tan}}^{-1}\left(\frac{r_{k+1}}{\sqrt{\sum_{k=1}^{Nc-1}{\left(r_{k}\right)}^{2}}}\right)$$ (3)

where *k* goes from 1 to (*N*_*c*_-1). This yields the transformed vector, **v**:

$$v=\left[\begin{array}{c} l\\ a_{1}\\ \vdots \\ a_{Nc-1} \end{array}\right]$$ (4)

Following this transformation, all the parameters in v become normally distributed.

Computation of mean and covariance matrices for each genotype (Training set only)

Transformed vectors of the same class or genotype from a training set were grouped together into a parameter matrix:

$$V_{i}=\left[\begin{array}{cccc} v_{1} & v_{2} & \cdots & v_{Ni} \end{array}\right]$$ (5)

where *N*_*i*_ is the number of melt curves in the training set for the *i* ^th^ class or genotype. *N*_*c*_ parameter matrices were be generated from the training set, one for each genotype. Thus the dimensions of each **V**_*i*_ are [*N*_*c*_ x *N*_*i*_]. (*N*_*c*_ *= 3* for all of the assays we tested)

For each class or genotype, the mean of each row of **V**_i_ and the covariance matrix of **V**_i_ were calculated to obtain the matrices **μ**_i_ and **C**_i_ respectively with dimensions [*N*_*c*_ x 1] and [*N*_*c*_ x *N*_*c*_] respectively.

### Genotype Classification for a DNA sample of unknown genotype

Once the mean and covariance matrices were obtained for each known genotype from a training set, a melt curve obtained from a DNA sample of unknown genotype was classified. As with the training set melt curve of known genotypes, each melt curve of unknown genotype in the validation set was transformed to a vector of spherical coordinates, **v**.

For the unknown DNA sample, the class-conditional density for each possible genotype was used to calculate the likelihood of **v** with respect to each possible genotype, *g*_*i*_ in Eq 6. The likelihood is a function of **v** and the mean and covariance matrices obtained from the training set according to the multivariate Gaussian distribution equation:

$$p\left(v|g_{i}\right)=\text{exp}\left(-\frac{1}{2}*{\left(v-\mu_{i}\right)}^{T}*{\left(C_{i}\right)}^{-1}*\left(v-\mu_{i}\right)-\frac{d}{2}*\text{log}\left(2\pi \right)-\frac{1}{2}*\text{log}\left(\left|C_{i}\right|\right)\right)$$ (6)

|**C**_*i*_| is the determinant of the matrix **C**_*i*_. *d* is the number of dimensions which is equal to the number of classes, *N*_*c*_, or 3 in our case.

The posterior probabilities (probability that the DNA is each of the possible genotypes) were calculated using Bayes’ Theorem. The class priors or frequencies of each genotype in the population in conjunction with the class-conditional densities were used to obtain the posterior probabilities:

$$p\left(g_{i}|v\right)=\frac{P\left(g_{i}\right)*p\left(v|g_{i}\right)}{\sum_{i=1}^{Ng}\left(P\left(g_{i}\right)*p\left(v|g_{i}\right)\right)}$$ (7)

where *p*(*g*_*i*_ | **v**) is the posterior probability for the *i* ^*th*^ genotype. The sum of all posterior probabilities adds up to 1. *P*(*g*_*i*_) is the class prior or the frequency of the *i* ^*th*^ genotype in the population. In this work, we assumed the frequency of each genotype in the population to be 1/3 each since all DNA genotypes were represented and their frequencies were not representative of the population.

The DNA sample is classified as the genotype with the largest posterior probability. If the genotype with the largest posterior probability is less than some acceptable threshold (i.e 99.5%) then a no call is given. Furthermore, if the vector **v** is not in contained within a certain ellipsoid containing the majority of points (ie. 99.99%) of the genotype with the largest posterior probability, a no call will result. This is calculated using the cumulative distribution of the multivariate normal distribution using the mean and covariance matrices obtained from the training set.

**Fig 3:**
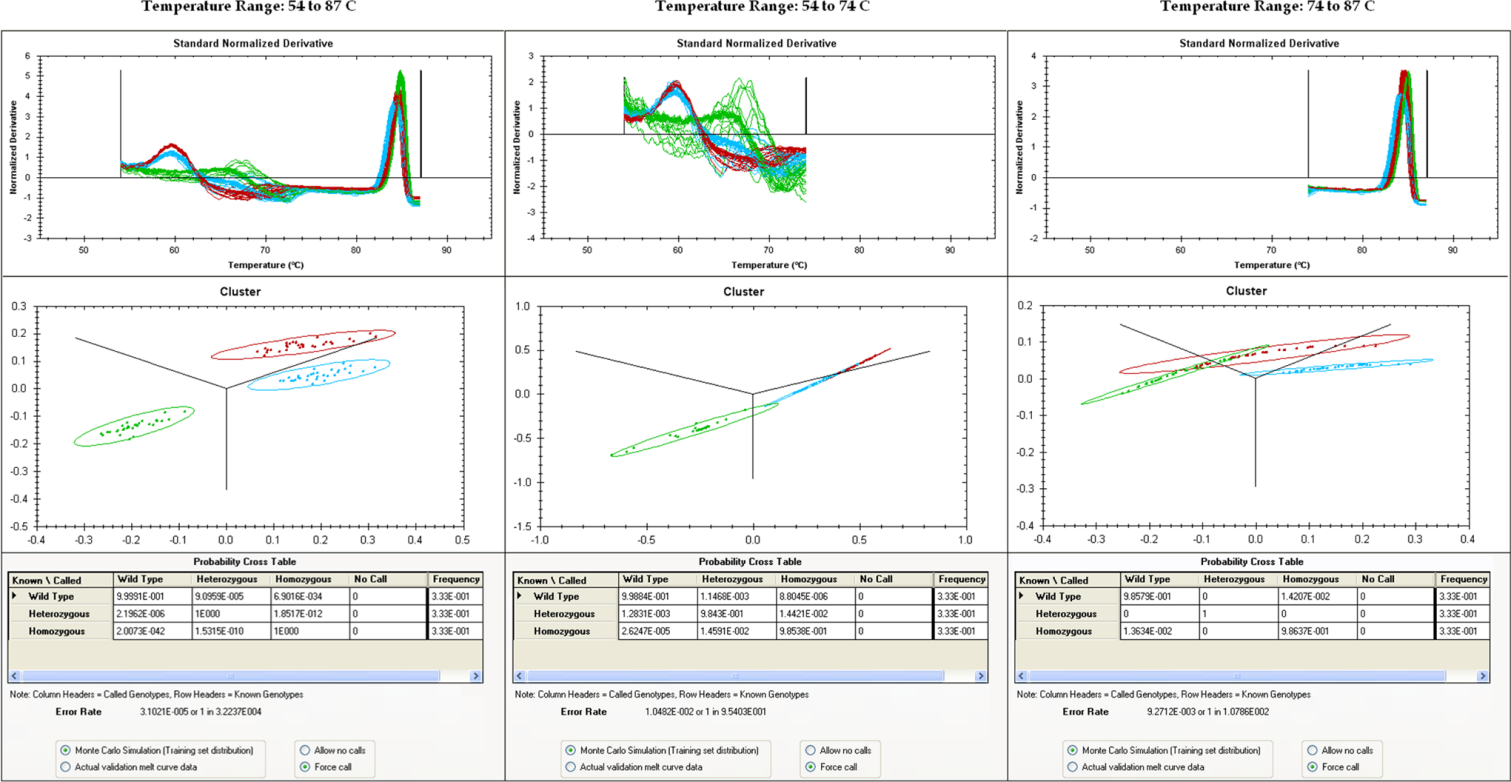
Separation of *MTHFR C*.*665C>T* genotypes using data from different temperature ranges. Left: 54 to 87C (probe and amplicon), middle: 54 to 74C (probe only) and right: 74 to 87C (amplicon only). Top: normalized derivative curves, middle: separation of genotype clusters in 2D. Bottom row shows the expected probability cross table via Monte Carlo simulation of 3D spherical coordinates.

Fig 3 demonstrates the optimal temperature range for correlation analysis in the automated genotyping procedure for the MTHFR c.665C>T assay. In this case the temperature range from 54 to 87°C was optimal in minimizing the estimated misclassification rate. The visualization of melt curves as clusters of 2D points is also described in the Text along with the ellipses that describe genotype distribution of the training set. A flowchart demonstrating the procedure by which the ellipses are generated from the training set is shown in S4 Fig. The procedure by which each melt curve is transformed to a 2D point is shown in S5 Fig. Visualizations of more than 3 genotype clusters is possible, however the user will have to select which 3 ANCKG genotypes to correlate against that determine the projected tri-axes.

## Results

The cross-validation genotype call of the blinded DNA samples yielded the results shown in Table 2. For the *VKORC1* and the CYP2C9*3 assays, all 120 blinded DNA melt curves were genotyped correctly. There is good separation among genotypes in the *VKORC1* and CYP2C9*3 assays as shown in Fig 2C & 2D and S6 Fig, respectively.

**Table 2:** Automated genotyping results.

Target	Full melt temp. range	Classification temp. range	Wild-type	Heterozygote mutant	Homozygote mutant
*VKORC1 c*.*1639G>A*	55°C–95°C	75°C–83°C	63/63	51/51	6/6
CYP2C9*2 c.*430C>T*	55°C–95°C	82°C–86°C	89/90^a^	18/18	10/12^b^
CYP2C9*3 c.*1075A>C*	55°C–95°C	74°C–82°C	87/87	30/30	3/3
*MTHFR c*.*665C>T*	45°C–95°C	54°C–87°C	75/75	48/48	24/24

^a,b^: For the CYP2C9*2 target assay, 1 wild-type sample and 2 homozygote samples were given no-calls due to maximum posterior probability values being less than 99.5%

**Fig 4:**
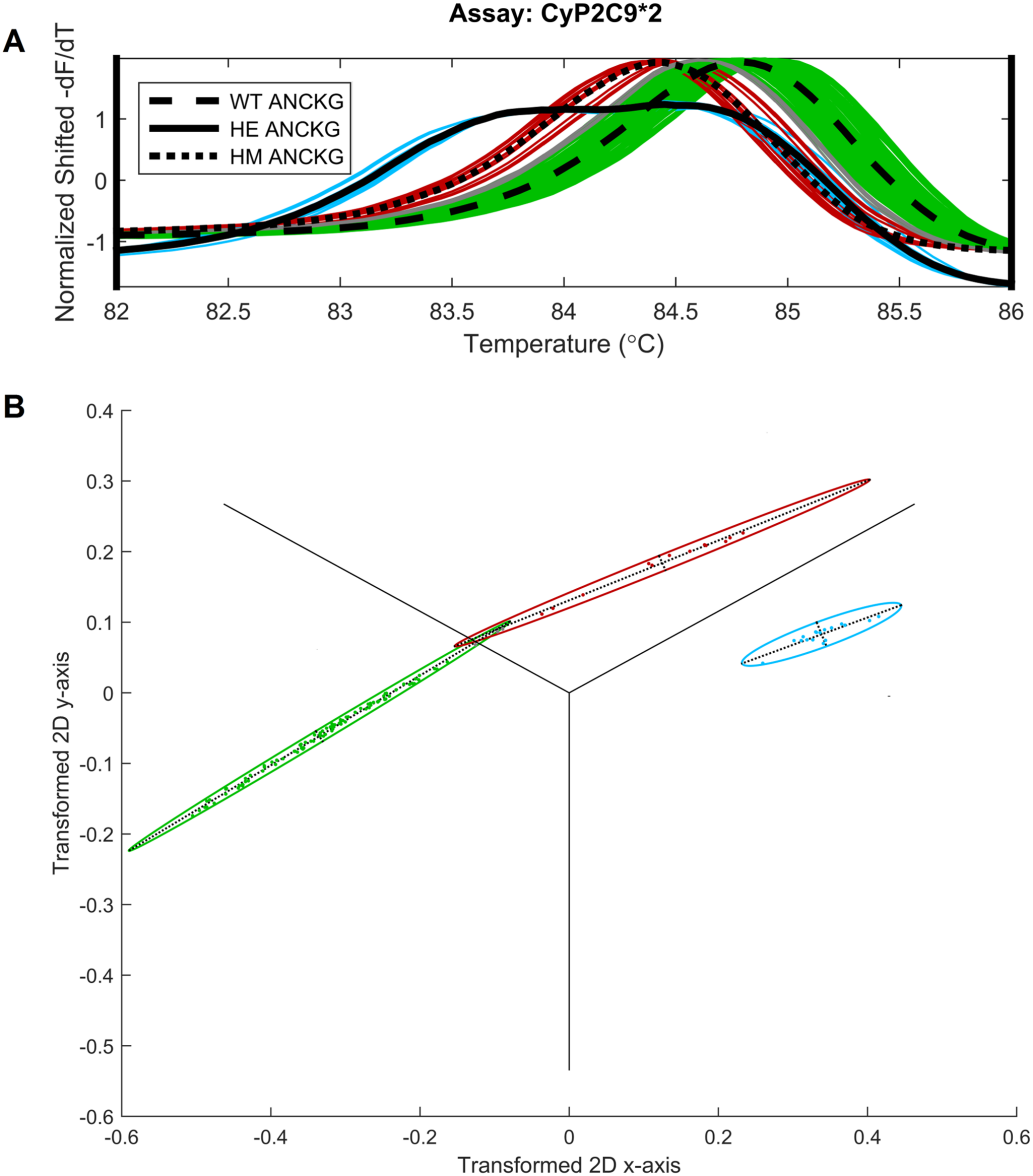
Cross validation set of the DNA samples of the CYP2C9*2 assay. A. Normalized shifted derivative curves. Colors denote the called genotype (green: wild-type, red: homozygous mutant, blue: heterozygous mutant). Curves colored in gray are given a “no call” B. Corresponding 2D transformation scatter plot for visualization purposes. The three gray points in the space between the wild-type and homozygous mutant ellipses are the points that correspond to the “no-call” melt curves.

For the CYP2C9*2 assay, 3 of the 120 DNA melt curves were given a no-call since their largest posterior probability values did not exceed 99.5%. Two of the three no call genotypes were actually homozygous mutants (that originated from the same patient sample placed in neighboring wells) and one was actually wild-type. The reason for the no call can be visualized in Fig 4. The identification of heterozygous DNA melt curves are clearly separate from the other two genotypes. However, because of some overlap between wild-type and homozygous mutant genotypes and the large variability of melt curves within each genotype, no calls are made by the software when melt curves fall between the two.

For the *MTHFR c*.*665C>T* locus, all 147 melt curves were genotyped correctly.

## Discussion

HRM was introduced in 2003 as a fast and effective way of genotyping [4,15], yet the classification of genotypes on currently marketed PCR and HRM instruments still requires a level of interaction by a trained user. The genotype call may vary depending on certain settings or parameters chosen by the user. With our software, the genotyping is fully automated, not requiring any user interaction with the data. In a high throughput lab or a point of care environment, user interpretation of melt curves should not be required. In serial PCR and melt instruments, particularly ones with reflexive testing where the result of one assay dictates the next assay to run, users need to be kept out of the loop.

Historically, differences in the melting temperature, *T*_*M*_ approximated by location of derivative peaks have been used to separate wild-type and homozygous mutants, and the presence of a second peak causing a shape change is used to identify heterozygous mutants [4,15]. There is more to a melt curve signature than a single *T*_*M*_ value that may enable us to differentiate homozygotes [2,9]. Melt data can be fit to thermodynamic models through nonlinear least squares where the sum squared error (SSE) between the model and data is minimized. Here enthalpy, florescence amplitude, a decay constant and a fluorescence offset in addition to *T*_*M*_ are identifiable parameters that can fully describe the dynamic region of a melt curve [16,17]. However, model equations and number of parameters vary depending on the genotype, or number of features. Furthermore, resulting fit parameters may not be ones that yield the lowest SSE, and the process can be slow due to its iterative nature. We made no model assumption in the method described here.

We decided to use the correlation coefficient of the derivative of a large segment of melt curve with respect to average melt curves of each possible known genotype as a quantifier. The software also statistically determines the probability that an unknown DNA sample belongs to each of the possible known genotypes. In the methods described to calculate posterior probabilities for genotyping or to estimate the misclassification rate by Monte Carlo simulation, the number of parameters is equal to the number of classes or genotypes which is 3 in the each of the four assays here. The same computation methods apply in multiplexing cases where the number of classes of melt curves can exceed 3. HRM curves of all unknown DNA genotypes are treated the same regardless of shape, number of peaks or features in the derivative of the melt curve.

As others have done, we have also shown temperature shifting necessary to account and correct for inter-plate temperature gradients as shown in Fig 2. This form of temperature shifting preserves the change in melting temperature between different genotypes, but controls for temperature measurement variability from run to run. Lower variance in parameters for samples within a genotype may be obtained by performing temperature shifts to control for temperature measurement bias and variability across the well plate (intra-plate). *T*_*M*_ bias as a function of well plate location has been demonstrated in the past as the temperature in all wells are measured by a single temperature sensor [6,18,19]. A unique temperature shift can be calculated per DNA sample by populating multiple positive controls around each sample in checkerboard fashion. Alternatively, internal temperature controls can be used in each assay where complimentary oligonucleotides create an additional peak at a precise known temperature far from the melt region for each DNA sample [20,21]. In the latter case, the temperature region used for temperature shifting is different than the melt region used for genotyping.

Vertical normalization helps bring melt curves of the same genotype close together while separating those from different ones. In the past, normalization techniques were used to remove the monotonic decaying background fluorescence not associated with the dissociation of DNA strands from the melt. After this process, fluorescence values start at 100% and end at 0% and the derivatives of fluorescence start and end at 0 before and after the melt. Although separating these two components of fluorescence is useful when using simulated mathematical thermodynamic models to predict experimental ones based on assay design and experimental conditions [22,23], we found simple scaling (with slope and intercept) to have zero mean and unit standard deviation within a defined temperature range sufficient for the purpose of automated genotyping. Training data and validation data (blinded) have to be obtained from the same type of instrument run under the same conditions. Scaling a signal does not alter its correlation with another signal; however it does ensure that each curve in the training set is weighed appropriately when calculating the ANCKG curves. To fully automate the genotyping procedure, rather than have the user select the temperature range for genotyping, we developed a Monte Carlo simulation procedure to automatically determine the range whereby the estimated misclassification rate is minimized (see S1 Text). This quantifier is also useful in providing feedback to engineers and scientists developing new instruments and assays for genotyping. Due to the overlap between CYP2C9*2 wild-type and homozygous mutant genotypes demonstrated in Fig 4, and the three no-calls in Table 2 the CYP2C9*2 assay formulation has since been modified to increase the separation of homozygous mutant and wild-type genotypes through the introduction of unlabeled probes.

Supervised classification of HRM curves have been done before by a few researchers using some form of Principal Component Analysis (PCA), Linear Discriminant Analysis (LDA) [12], or Supervised Vector Machine (SVM) learning [24], however the parameters derived from melt curves and methods are different from what we describe.

LDA requires the assumption that the distributions of clusters for all genotypes have the same covariance matrix in determining the posterior probabilities. In our methods, no such assumption is required as it is not only conceivable, but likely that the covariance matrices of different genotypes are quite different demonstrated by the different sizes and orientations of ellipsoids and ellipses. With SVM, the distribution of parameters can be arbitrary (not defined by an equation), however this makes the computation of likelihoods and probabilities associated with each genotype call difficult. Also, SVM is only directly applicable for classification between exactly two genotypes, so multiple paired comparisons are required.

Any automated genotyping procedure requires that each dynamic melt curve be transformed into a vector consisting of a finite number of parameters prior to employing the myriad of classification techniques. The novelty in our procedure is in how we transform a dynamic melt curve (fluorescence versus temperature profile) into a limited parameter set whereby each of the parameters are normally distributed. For each melt curve, correlation values with respect to each ANCKG are transformed to a spherical parameters, each of which are normally distributed shown by S1 Fig. This allows us to use the multivariate normal equation to calculate likelihoods and posterior probabilities for each possible genotype.Our method is able to classify any number of genotypes >1 where the number of parameters used to represent each melt curve for classification is equal to the number of possible genotypes.

There are certain prerequisites and limitations to our classification procedure. Because we make no mathematical model assumptions to what form each dynamic melt curve takes, an ample training set of melt curves of known genotypes is required such that the estimated mean and covariance matrices for each genotype match that of the general population. For the procedure to work, the reagents and run conditions of the training set have to match those of the unknown samples. When melt curves between different genotypes are close (they overlap throughout the entire temperature range) such that the genotype with the largest posterior probability does not exceed a predefined threshold, the algorithm does not make a call, however, the algorithm automatically decides on what’s to close to call based on this pre-defined acceptable threshold. The threshold is predetermined by a clinical expert who weighs the risks of a no-call versus an inaccurate result for that particular assay given their frequencies of occurrence.

Alternative transformation of the original correlation parameters from 3D to 2D provides a visual cluster representation of melt curves of different genotypes in an intuitive manner. Ellipses are generated from the training set, and each point represents a unique melt curve. The triaxis define the maximum correlation boundaries between the point and the ANCKG of each of the three genotypes. Although atypical, genotypes with the highest correlation and highest posterior probability can differ when a point representing a sample is contained within the edge of an ellipse that crosses over the one of the three axes. The identification of a melt curve whose parameterized point representation is far away from training set distributions of known genotypes represented by their ellipses and ellipsoids may be attributed to a new genetic variant never seen before.

In conclusion, we have devised a machine learning algorithm to fully automate genotyping of HRM melt curves without user interpretation. We also devised a way to visualize genotype clusters and distributions as 2D points and ellipses relative to an intuitive coordinate system whose boundaries define regions of maximal correlation. Lastly we devised a Monte Carlo simulation method that yields an estimate of misclassification rate. This quantifier is important as it allows the software to automatically determine optimal parameters such as temperature range in which to perform analysis. It also gives feedback to the assay scientists and engineers on the expected performance of the system through multiple iterations of development.

## Supporting Information

S1 Fig Flowchart of classification training procedure. (TIF)

S2 Fig Flowchart of validation (genotyping) procedure. (TIF)

S3 Fig Distribution of correlation coefficients of a group of wild-type normalized shifted derivative curves against those of the A. average wild type, B. average heterozygous mutant, and C. average homozygous mutant. The bottom plots show that following a transformation to spherical coordinates (D, E and F) all parameters are normally distributed. Shapiro-Wilk normality test p-values are listed.
(TIF)

S4 Fig Flowchart of visualization of training set as 2D ellipses. (TIF)

S5 Fig Visualization of each melt curve as a point in 2D space. (TIF)

S6 Fig Separation of CYP2C9*3 genotypes A. Shifted, normalized–*dF/dT* curves with training set genotype averages (black lines) B. Corresponding 2D scatter plot. (TIF)

S1 Table Posterior probability cross table. (DOCX)

S1 Text Optimal temperature range determination and quantification of misclassification rate. (DOCX)

S2 Text Visualization of High Resolution Melt curves as 2D clusters. (DOCX)

## Abbreviations

HRM: High Resolution Melting
PCR: Polymerase Chain Reaction
ANCKG: Averaged Normalized Curve of Known Genotype
*T_M_*: Melting Temperature
