# Supplementary figures and images for: Automated Classification and Cluster Visualization of Genotypes Derived from High Resolution Melt Curves
Source: PLoS One. 2015 Nov 25;10(11):e0143295. doi: 10.1371/journal.pone.0143295 (PMC4659556; doi:10.1371/journal.pone.0143295)

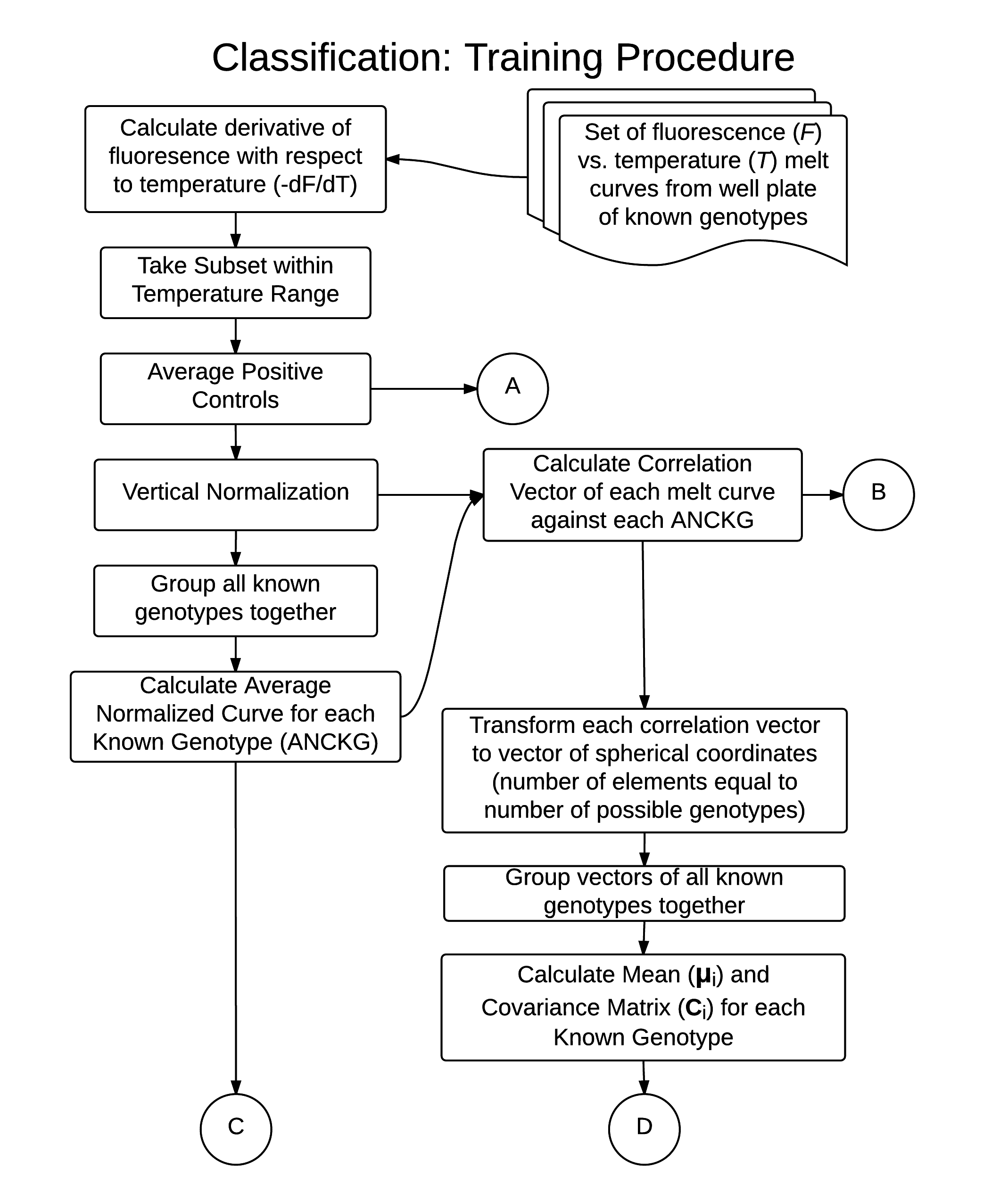

Supplement: S1 Fig — (TIF) [file pone.0143295.s001.tif]

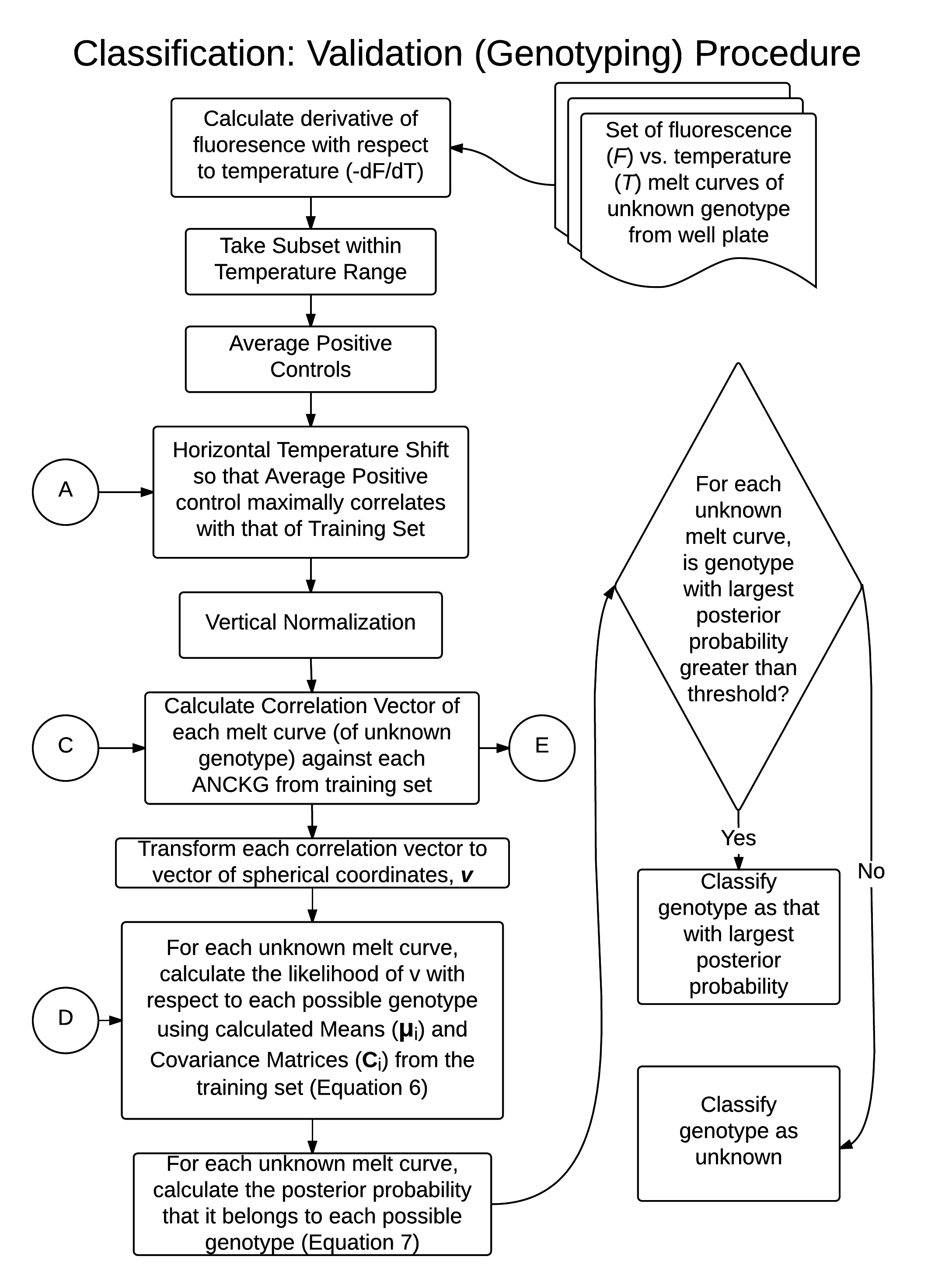

Supplement: S2 Fig — (TIF) [file pone.0143295.s002.tif]

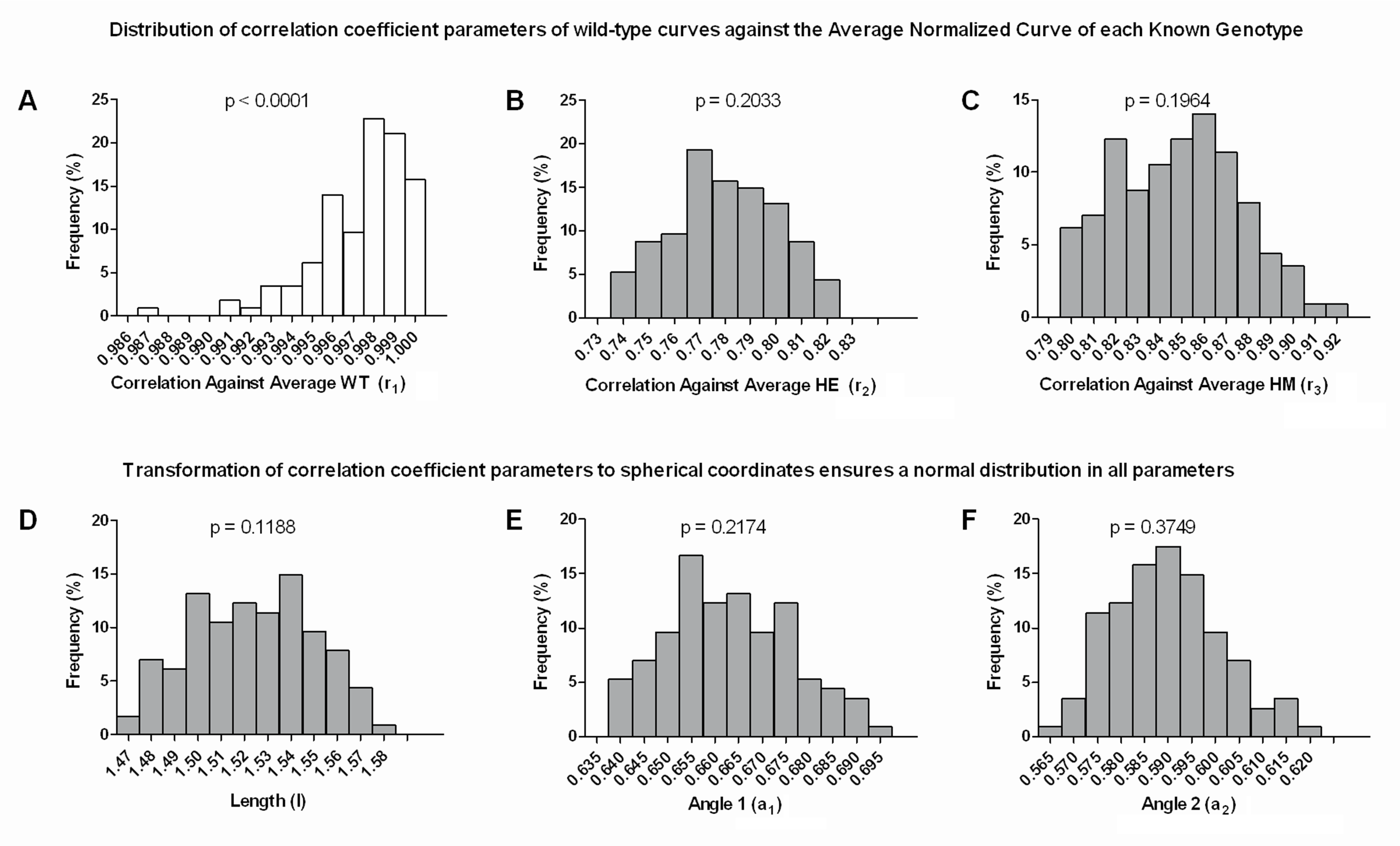

Supplement: S3 Fig — Distribution of correlation coefficients of a group of wild-type normalized shifted derivative curves against those of the A. average wild type, B. average heterozygous mutant, and C. average homozygous mutant. The bottom plots show that following a transformation to spherical coordinates (D, E and F) all parameters are normally distributed. Shapiro-Wilk normality test p-values are listed. (TIF) [file pone.0143295.s003.tif]

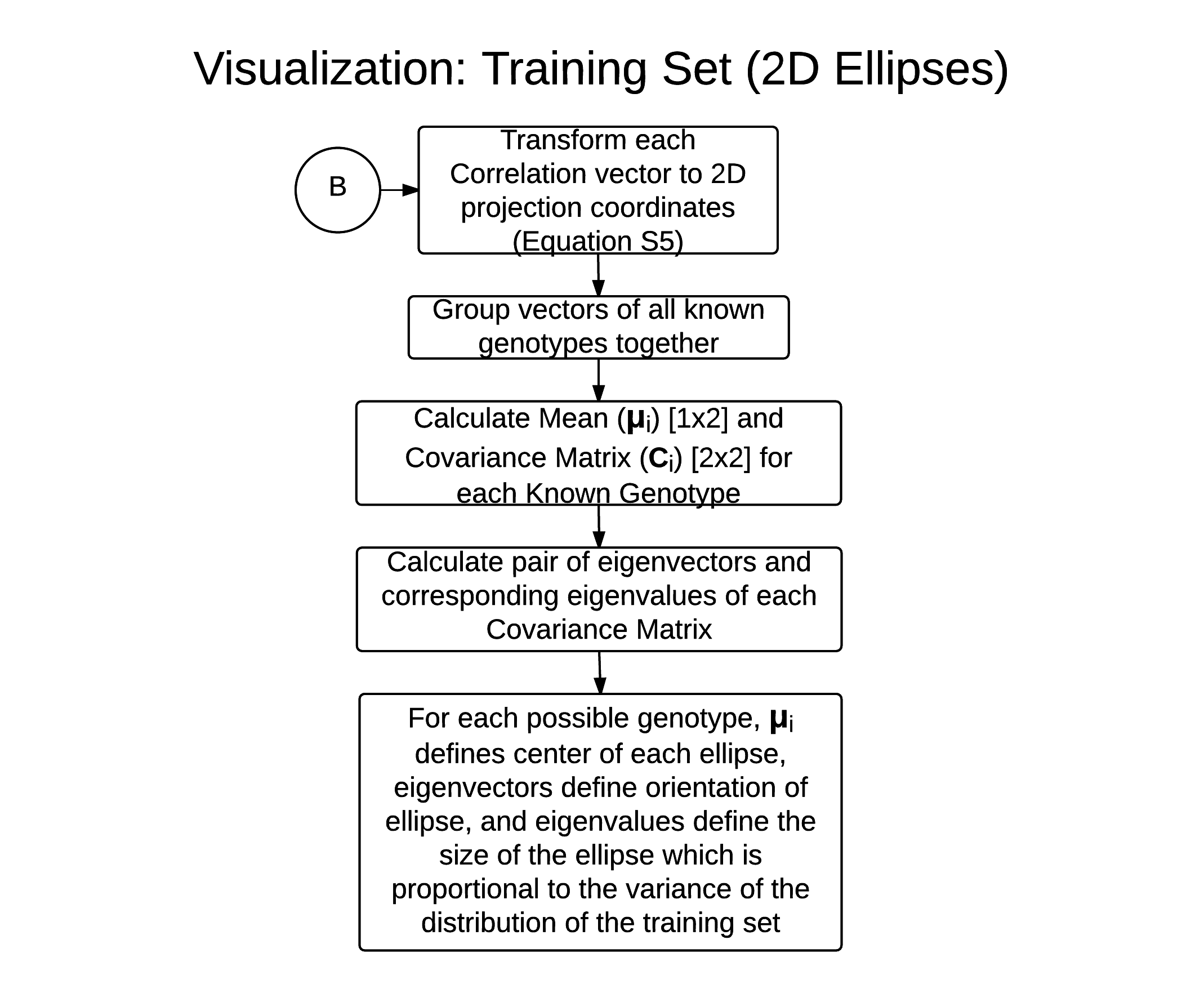

Supplement: S4 Fig — (TIF) [file pone.0143295.s004.tif]

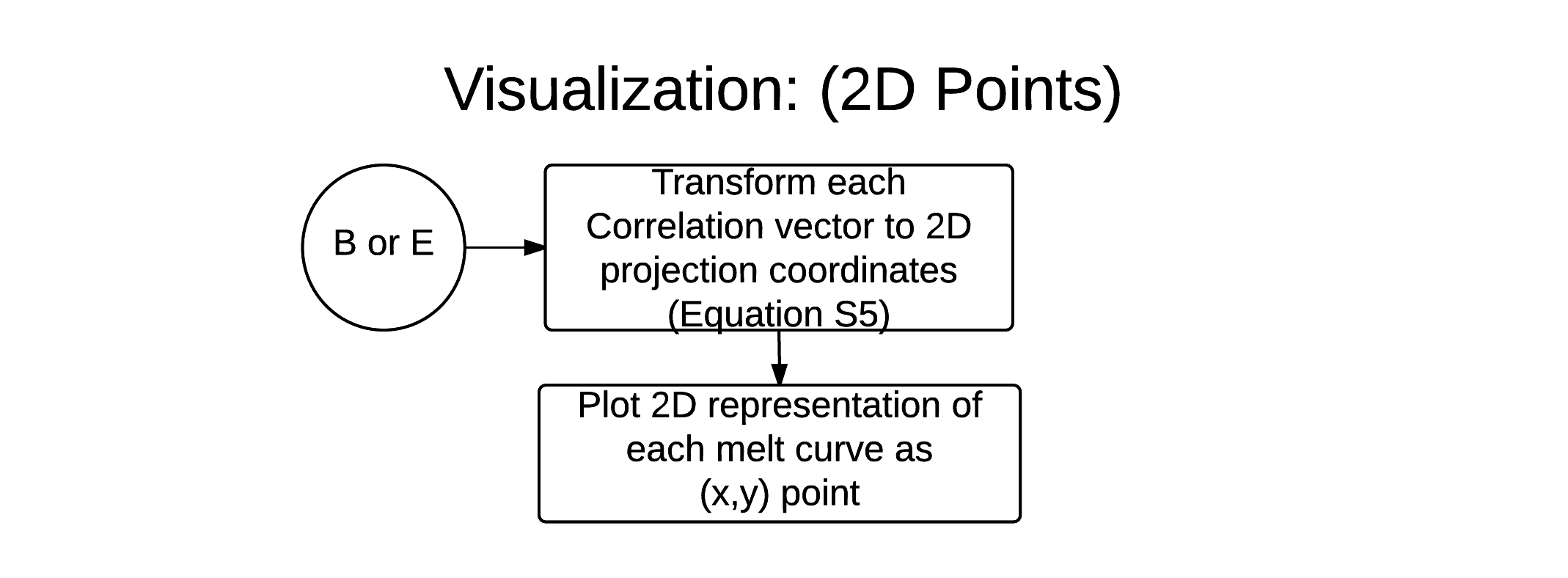

Supplement: S5 Fig — (TIF) [file pone.0143295.s005.tif]

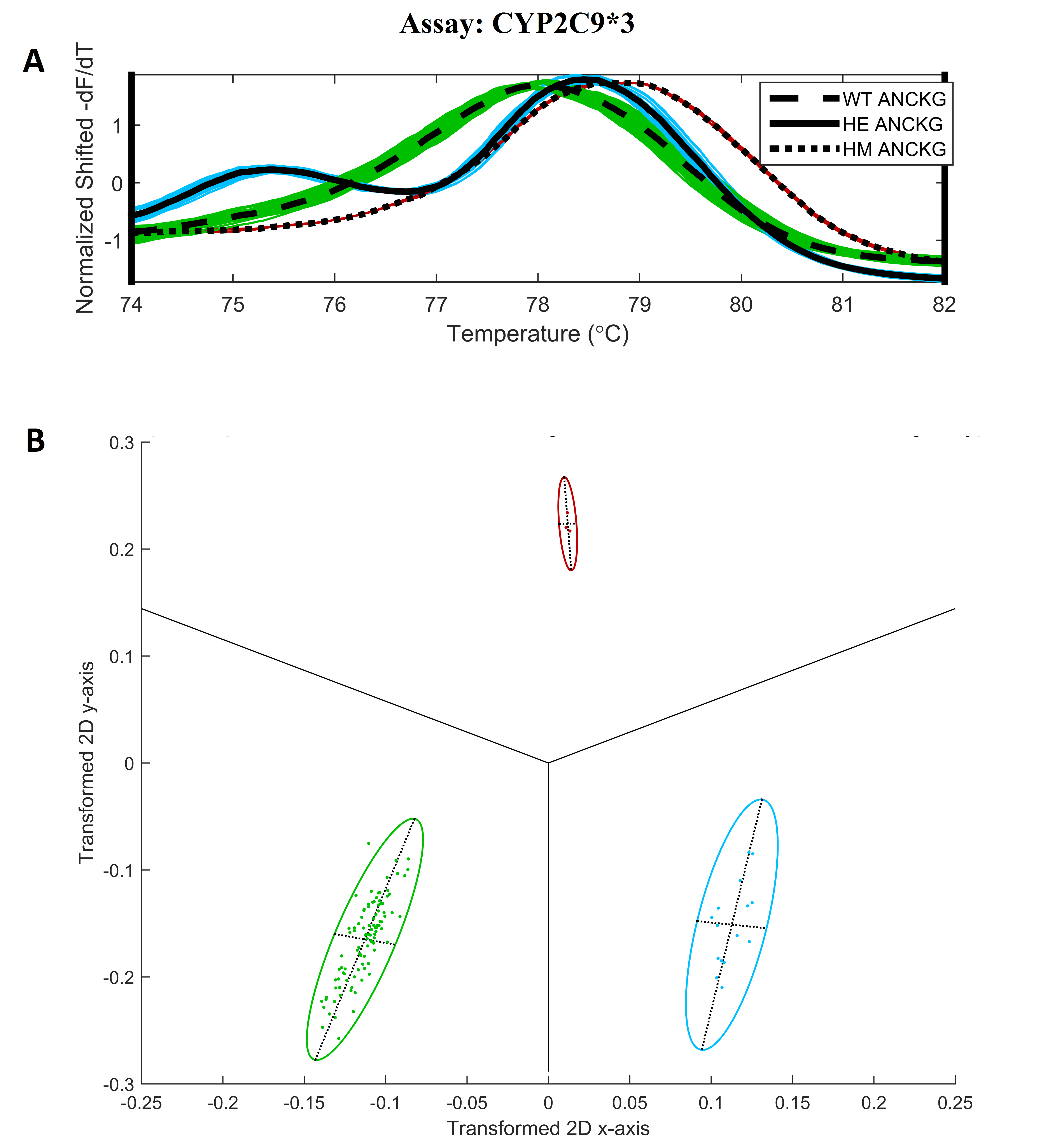

Supplement: S6 Fig — (TIF) [file pone.0143295.s006.tif]
