# Supplementary material for: Automated Classification and Cluster Visualization of Genotypes Derived from High Resolution Melt Curves
Source: PLoS One. 2015 Nov 25;10(11):e0143295. doi: 10.1371/journal.pone.0143295 (PMC4659556; doi:10.1371/journal.pone.0143295)
Supplement: S1 Table — (DOCX) [file pone.0143295.s007.docx]

**S1 Table:** Posterior probability cross table

| **↓ Known Genotype** (rows) **/**  **→ Called Genotype** (cols) | **Wild-type** | **Heterozygous** | **Homozygous** |
| --- | --- | --- | --- |
| **Wild-type** | $\bar{p}\left(g_{WT}\vert\mathbf{v}_{WT}\right)$ | $\bar{p}\left(g_{HE}\vert\mathbf{v}_{WT}\right)$ | $\bar{p}\left(g_{HM}\vert\mathbf{v}_{WT}\right)$ |
| **Heterozygous** | $\bar{p}\left(g_{WT}\vert\mathbf{v}_{HE}\right)$ | $\bar{p}\left(g_{HE}\vert\mathbf{v}_{HE}\right)$ | $\bar{p}\left(g_{HM}\vert\mathbf{v}_{HE}\right)$ |
| **Homozygous** | $\bar{p}\left(g_{WT}\vert\mathbf{v}_{HM}\right)$ | $\bar{p}\left(g_{HE}\vert\mathbf{v}_{HM}\right)$ | $\bar{p}\left(g_{HM}\vert\mathbf{v}_{HM}\right)$ |
