# Supplementary material for: Automated Classification and Cluster Visualization of Genotypes Derived from High Resolution Melt Curves
Source: PLoS One. 2015 Nov 25;10(11):e0143295. doi: 10.1371/journal.pone.0143295 (PMC4659556; doi:10.1371/journal.pone.0143295)
Supplement: S1 Text — (DOCX) [file pone.0143295.s008.docx]

**S1 Text: Optimal temperature range determination and quantification of misclassification rate**

As stated in the manuscript only a subset of the derivative of the fluorescence curve is used in the correlation analysis. It is best to use data within a temperature range that maximally separates the genotypes of 3D clusters (in spherical coordinates), specifically to minimize the error rate of the genotype call. Fischer derived the ratio of the between (genotype) class scatter to the within class scatter as a quantifier to maximize for use in Linear Discriminate Analysis. Although this quantifier has a direct analytical solution, it is based on the assumption that the covariance matrices of the different genotypes derived in Fig. S1 are the same which is not true in our case. Furthermore, we specifically wish to minimize the quantifier that is the error or misclassification rate. For that reason we used enhanced Monte Carlo Simulations taking advantage of multivariate normal distributions to estimate the misclassification rate derived from the training set. For each *i*^th^ genotype cluster with mean and covariance matrices μ_i_ ([*N_c_* x 1]) and $\mathbf{C}_{i}$([*N_c_* x *N_c_*])) derived from the training set, additional data points can be sampled at random with the same distribution as follows:

, (S1)

*N_c_* is the number of classes or genotypes in the training set which is 3. The function $randn(N_{c},1)$ creates a [3 x 1] vector whereby when sampled many times has a mean of 0 and standard deviation of 1. $Chol(\mathbf{C}_{i})$ is the Cholesky factorization of the covariance matrix $\mathbf{C}_{i}$that when applied in Equation S1 yields a set of points with the same distribution as the training set for each genotype. 10000 random points are sampled for each known genotype and the posterior probability value is calculated for each sampled point. These values are averaged over the 10000 points and tabulated in S1 Table.

Ideally, the diagonals of this probability cross table should be 1 and the off diagonals should be 0. For any known genotype (row), the off diagonal terms in that row is the expected misclassification rate for that genotype as follows.

Wild-type misclassification rate: (S1)

Heterozygous misclassification rate: (S2)

Homozygous misclassification rate: (S3)

The overall expected misclassification rate of any DNA sample, of any genotype picked at random is a linear combination of misclassification rates of the individual genotypes scaled by their population frequencies (priors) as follows:

$err=P\left(WT\right)err_{WT}+P\left(HE\right)err_{HE}+P\left(HM\right)err_{HM}$ (S4)

The software iterates different temperature ranges at one degree increments to cover the full melt temperature range and recalculates the expected misclassification rate each time. The optimal temperature range selected is the one that yields the lowest expected misclassification rate.
