# Supplementary material for: Automated Classification and Cluster Visualization of Genotypes Derived from High Resolution Melt Curves
Source: PLoS One. 2015 Nov 25;10(11):e0143295. doi: 10.1371/journal.pone.0143295 (PMC4659556; doi:10.1371/journal.pone.0143295)
Supplement: S2 Text — (DOCX) [file pone.0143295.s009.docx]

**S2 Text: Visualization of High Resolution Melt curves as 2D clusters**

For classification, the number of parameters is equal to the number of classes, *N_c_*. The transformation to get from **r** to **v** for classification purposes preserves the number of dimensions and there is no information loss. Alternatively, a different transformation could have been used to reduce the number of dimensions from 3 to 2 as shown in S4 Fig. and S5 Fig.

$\mathbf{v}=\mathbf{T}*\mathbf{r}$ (S5)

where **T** is a [2x3] linear transformation matrix when multiplied with **r,** a [3x1] vector produces **v** that is a [2x1] vector. In this case there are 3 classes but a 2 parameter space where:

$\mathbf{T}=\left[\begin{matrix} -1 & 1 & 0 \\ -1/\sqrt{3} & -1/\sqrt{3} & 2/\sqrt{3} \end{matrix}\right]$ (S6)

Here again, as in Equation 5 of the manuscript, transformed vectors, **v** of the same class or *i*^th^ genotype from a training set are grouped together into a parameter matrix, **V**_i_ and the average and covariance matrix is calculated for each. The matrix **T** in is a projection of the correlation vector to a 2D plane with normal vector passing through (1,1,1). This yields a 2D visual representation of how well separated the different clusters representing different genotypes are. The three axes (triaxis) in this 2D plot at (30, 150 and 270 degrees) represent the boundaries where correlation of the dynamic melt curve represented by each data point is maximal against the average curves of known genotypes from the training set. Classification of the genotype of an unknown melt curve is not necessarily assigned to the maximum correlation value, but the genotype with the highest posterior probability as stated in the manuscript. The distribution of points in each genotype cluster can be represented by ellipses as shown if Figure 1H, 2B and 2D. Each ellipse represents the class conditional density for each genotype represented by the likelihood (Equation 6 of manuscript). In the 2D visualization case *d* is 2, and the dimensions of μ_i_ and **C**_i_ are [1x2] and [2x2] respectively which are obtained from the training set. The long axis and short axis of each ellipse are always perpendicular to each other, but their orientation or angle relative to the horizontal axis is determined by the eigenvectors of **C**_i_. The magnitude of the corresponding eigenvalues is the variance of the data along the eigenvector directions. For the 2D ellipse to cover +/- 3 standard deviations of the distribution of the cluster, then half the length of the major and minor axes axis should be$3\sqrt{\lambda_{2}}$, and $3\sqrt{\lambda_{1}}$ where $\lambda_{2}$ and $\lambda_{1}$are the larger and smaller eigenvalues. In this case, in theory each ellipse should contain 98.9% of the training set data which are the ellipse borders plotted in the manuscript.
